# It’s about time: small mammal communities and Lyme disease emergence

**DOI:** 10.1038/s41598-023-41901-z

**Published:** 2023-09-04

**Authors:** V. Millien, S. S. T. Leo, S. Turney, A. Gonzalez

**Affiliations:** 1 Redpath Museum, McGill University, Montréal, QC H3A 0C4 Canada; 2 Department of Biology, McGill University, Montréal, QC H3A 1B1 Canada

**Keywords:** Ecology, Climate-change ecology, Community ecology

## Abstract

Theory predicts that biodiversity changes due to climate warming can mediate the rate of disease emergence. The mechanisms linking biodiversity-disease relationships have been described both theoretically and empirically but remain poorly understood. We investigated the relations between host diversity and abundance and Lyme disease risk in southern Quebec, a region where Lyme disease is rapidly emerging. We found that both the abundance of small mammal hosts and the relative abundance of the tick’s natural host, the white-footed mouse (*Peromyscus leucopus*), influenced measures of disease risk in tick vectors (*Borrelia burgdorferi* infection abundance and prevalence in tick vectors). Our results suggest that the increase in Lyme disease risk is modulated by regional processes involving the abundance and composition of small mammal assemblages. However, the nature and strength of these relationships was dependent both on time and geographic area. The strong effect of *P. leucopus* abundance on disease risk we report here is of significant concern, as this competent host is predicted to increase in abundance and occurrence in the region, with the northern shift in the range of North American species under climate warming.

## Introduction

Lyme disease was first described as Lyme arthritis in 1976 in Lyme, Connecticut^1,2^ and by 1996, it had become the most common vector-borne disease in the United States^3^. More recently, Lyme disease has begun to emerge in southern Canada^4^, and its incidence has increased steadily over the past two decades^5^.

Lyme disease is a vector-borne zoonotic disease caused by members of the bacterial species complex *Borrelia burgdorferi* sensu lato, which is endemic to much of the Northern Hemisphere^6–9^. In eastern North America, the vector of the Lyme disease causing bacterium *B. burgdorferi* sensu stricto (*B. burgdorferi* s.s. hereafter) is the blacklegged tick, *Ixodes scapularis*, which has three parasitic stages in its life cycle: larva, nymph, and adult.

Larvae and nymphs tend to take blood meals from small to medium-sized mammals, as well as ground-feeding birds, while adult females feed almost exclusively on large mammals, primarily the white-tailed deer (*Odocoileus virginianus*)^8,10^. Tick hosts differ in their efficiency as *B. burgdorferi* s.s. reservoirs, measured by the persistence of transmissible infection and the proportion of ticks that acquire infection after feeding upon infected host individuals. White-footed mice (*Peromyscus leucopus*) are particularly competent hosts: infected individuals may exhibit lifelong infectivity—although the duration is strain-dependent^11–13^—and can transmit infection to a very high proportion of feeding ticks^14^. Other common small mammal hosts include species of rodents and shrews that vary greatly in their competence as reservoir for *B. burgdorferi* s.s., from about 30% to over 50%^10^.

A number of host and vector species are involved in Lyme disease epidemiology, and the nature of the relationship between biodiversity and Lyme disease prevalence is debated^10,15–23^. Ostfeld and Keesing^24^ hypothesized that increased host diversity decreases the relative abundance of white-footed mice (the most effective reservoir) in host communities, leading to reduced infection prevalence, and therefore reduced disease risk^24,25^. They referred to this hypothesis as a “dilution effect”, although the term has come to be used more generally to refer to any negative relationship between diversity and disease risk, regardless of the mechanism^26^. More specifically, this reduction in infection prevalence via “wasted bites” of ticks on hosts other than white-footed mice is called frequency-dependent dilution^27^. Another potential dilution effect mechanism is density-dependent dilution. In this case, increased host diversity may decrease the absolute abundance of white-footed mice via competition, leading to a decrease in tick abundance and the abundance of ticks infected with *B. burgdorferi* s.s. For a dilution effect to occur, the most competent species, here the white-footed mouse, must also be the most common one, so that pathogen circulation is maintained even in the most species-poor host communities; this outcome results in a nested structure of the data matrix describing the occurence of several host species across sites (site x host species matrix), whereby one or a few host species are found at all sites, while the others are found across a subset of the sites^28^. Alternatively, because *I. scapularis* exploits a wide range of hosts, increased host diversity may instead increase the overall density of competent hosts, which could lead to increased tick density, and therefore increased disease risk^26,29,30^. Such a positive relationship between biodiversity and disease is known as an “amplification effect” (reviewed in^31^), and as with the dilution effect, a number of mechanisms may drive this relationship^26^. Recently, Keesing and Ostfeld^32^ argued that the apparent paradox between the two opposing effects of biodiversity on zoonotic diseases can be explained when distinguishing between natural biodiversity and the ongoing global biodiversity loss.

Long-term longitudinal data are essential to better characterize the dynamics of the relation between host diversity and zoonotic diseases risk, and to develop predictive models to anticipate and mitigate this risk. However few studies on the relationship between host diversity and *B. burgdorferi* s.s. prevalence have taken place in southern Canada, where Lyme disease is emerging. A field study in Quebec found that the overall nymphal abundance increased with small mammal species richness^33^. At a much finer spatial scale (approximately 2 sq. km), Dumas et al.^34^ did not detect any relation between host diversity and tick abundance, although in this study, pathogen prevalence was not evaluated. Another field study in Ontario found an overall negative correlation between the number of infected nymphs and small mammal species richness, with a modulating effect of white-footed mouse abundance^35^, which suggests a dilution effect.

Underlying the rapid emergence of Lyme disease in southern Canada is the northward expansion of the range of *I. scapularis* ticks^36–38^ followed by *B. burgdorferi* s.s.^9,39–41^, while northward expansion of the range of highly efficient white-footed mouse reservoirs accentuates the geographic footprint of Lyme disease risk in Quebec^40,42^. Significantly, there is evidence that range expansion of both ticks and white-footed mice is being facilitated by climate change^37,40–47^. Therefore, the emergence of Lyme disease in southern Quebec provides an important observatory to study how host diversity mediates the emergence of zoonotic disease via climate change-driven invasion into wildlife host communities.

**Figure 1:**
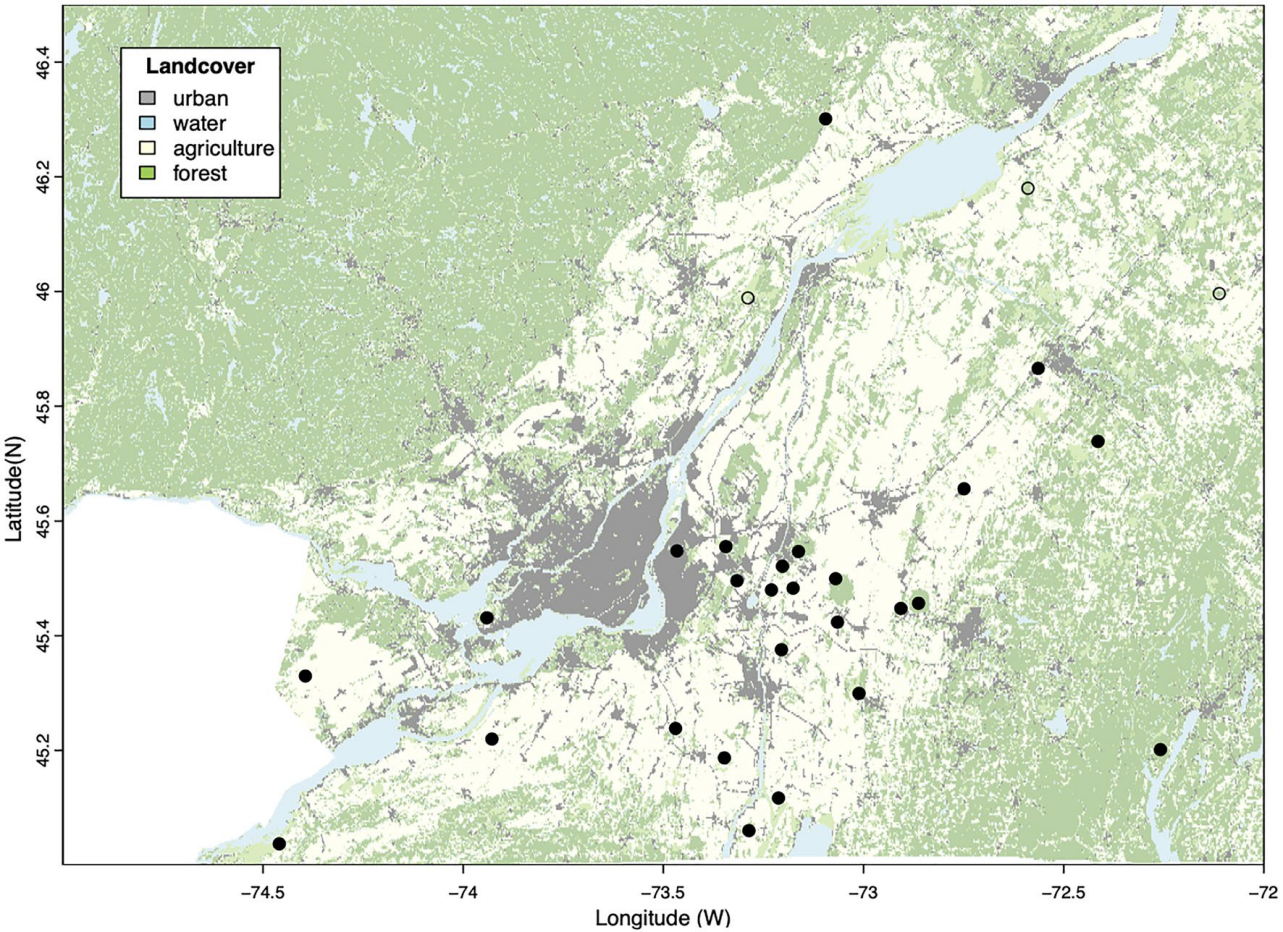
Map of the distribution of forest fragments and the surrounding land cover where sampling occurred in Southern Quebec (see Table S1 for details). The white-footed mouse (*Peromyscus leucopus*) was present (filled circles) at all study sites but three site (open circles) in the northeast of the study area.

Here, we sampled small mammals and ticks from 29 forest patches from 2011 to 2014 in southern Quebec and tested them for *B. burgdorferi* s.s. (Fig. 1)*.* We determined the relationship between small mammal diversity and abundance and the abundance and prevalence of infected ticks at our sites, while accounting for the effect of time (year) and geographic location in southern Quebec. We found evidence for both amplification and dilution effects occurring jointly across our study area, and the net effect of small mammal host community composition and abundance on pathogen prevalence varied locally, a variation we attribute to the distinct degree of establishment of tick populations and disease emergence in the region.

## Results

### Small mammal hosts and tick vectors

We conducted tick-dragging at all 29 study sites and searched for feeding ticks on all small mammals we collected. Ticks were not detected at 4 of the 29 sites (Table S1). Only data for *I. scapularis* were included in our analyses, but we also occasionally collected ticks of other species including *Dermacentor* sp., *Haemaphysalis leporispalustris, I. angustus, I. cookei, I. marxi,* and *I. muris*. In total, we collected 2877 *I. scapularis*, including 26 adults, 381 nymphs and 2470 larvae (representing 427 pools of larvae). The majority of sampled ticks were pools of larvae (51.2%) and nymphs (45.7%), while adults were rare (3.1%). Of these ticks, 468 (56.1%) were questing and 366 (43.8%) were feeding on hosts. There was no seasonal variation in the number of nymphs or larvae sampled (effect of month of collection: all *p* > 0.05). Overall, none of the host species fed significantly more ticks than other host species (*F* = 2.02, *p* = 0.052), but the maximum tick burden in a given host individual was observed in *P. leucopus* (113 ticks). Other hosts fed a maximum of 65, 27, 23 and 21 ticks (*B. brevicauda*, *N. insignis*, *P. maniculatus* and *T. striatus*, respectively), or less than 10 ticks (*S. cinereus*, *M. gapperi* and *Z. hudsonius*). Finally, there was no effect of sampling method on the abundance or prevalence of infected ticks. The abundance of feeding ticks was positively correlated with the abundance of questing ticks at a site (*R* = 0.68, *p* < 0.0001), and so was the prevalence of infected feeding ticks with the prevalence of infected questing ticks (*R* = 0.58, *p* < 0.0001). We thus pooled the feeding and questing tick data and used the total abundance of ticks and the total prevalence of infected ticks in further analyses.

A total of 1222 individuals of 9 small mammal species were trapped at the 29 sites, and the most abundant species was *P. leucopus* (*n* = 711, Table S2). The other species occurring at our sampling sites, in order from most to fewest number trapped were *Myodes gapperi, Blarina brevicauda*, *Sorex cinereus, Napaeozapus insignis*, *Tamias striatus*, *Zapus hudsonius* and *Sorex fumeus* (Table S2). The number of species captured at a site ranged from 1 to 7 (*average* = 3.82, *SD* = 1.33). The white-footed mouse was present at all sites, except for three sites in the northeastern area of the study region (Table S1, Fig. 1)*.* At sites where the white-footed mouse was present (with a few exceptions), more white-footed mice were trapped than any other small mammal. The community matrix temperature for all 29 sites was 18.43 and only 31.1% of the random community matrices had a lower temperature value, or were more nested than the observed community matrix (Fig. S1). The most common host species across our sites were *P. leucopus*, *B. brevicauda*, *M. gapperi* and *P. maniculatus* (26, 27, 15 and 17 out of 29 communities, respectively) and host abundance increased with the number of host species (*R* = 0.35, *p* < 0.006). Overall, the relative abundance of white-footed mice decreased with the number of small mammal species (*R* = − 0.56, *p* < 0.0001), but the absolute abundance of white-footed mice did not (*R* = − 0.11, *p* = 0.41).

### Infection prevalence with *Borrelia burgdorferi*

*B. burgdorferi* s.s. infection of ticks was detected at 11 sites (44% of the sites where ticks occurred). *B. burgdorferi* s.s. was not present at the sites where *P. leucopus* was absent. In total, we collected 8 infected adults (all questing), 79 infected nymphs (59 questing and 20 feeding) and 29 pools of feeding larvae in which *B. burgdorferi* s.s. was detected (representing a maximum of 209 larvae). A majority of the infected ticks were collected from dragging (57.8%, Table S1), especially for nymphs (74.7%) and adult ticks (100%). Infected larvae were all collected on small mammal hosts (questing larvae were not tested and were assumed to be free of *B. burgdorferi* s.s.). Overall, 29 (70.7%) pools of feeding larvae, 20 (25%) feeding nymphs, and 59 (19.6%) questing nymphs were infected. Infected feeding ticks were collected on seven distinct host species representing 36 individuals: *P. leucopus* (24 individuals), *P. maniculatus* (5), *T. striatus* (2), *M. gapperi* (2), *B. brevicauda*, *N. insignis* and *Sorex cinereus* (1 individual each). DNA of *B. burgdorferi* s.s. was detected in tissues of only five individuals of these hosts, all of which were *P. leucopus*.

### Preliminary analyses

The geographic distance between our study sites ranged from 35 to 212 km and we detected a significant spatial autocorrelation in most of the mammal hosts and tick vector data (Fig. S2). We thus included the geographic coordinates of each site in further analyses.

We then evaluated the correlation between the four measures of mammal host at each site (number of host species, abundance of hosts corrected for sampling effort, abundance of white-footed mice corrected for sampling effort, and relative abundance of white-footed mouse). The number of mammal host species was significantly correlated with the white-footed mouse relative abundance (*R* = − 0.62, *p* < 0.0001), while the abundance of white-footed mice was correlated with the number and abundance of mammal host species (*R* = − 0.42, *p* < 0.0001 and *R* = 0.47, *p* < 0.0001, respectively), as well as with its relative abundance (*R* = 0.65, *p* < 0.0001). We thus opted to retain only two variables in further analyses to avoid collinearity: the abundance of small mammal hosts and the relative abundance of white-footed mice.

### Small mammal hosts and abundance of infected tick vectors

**Figure 2:**
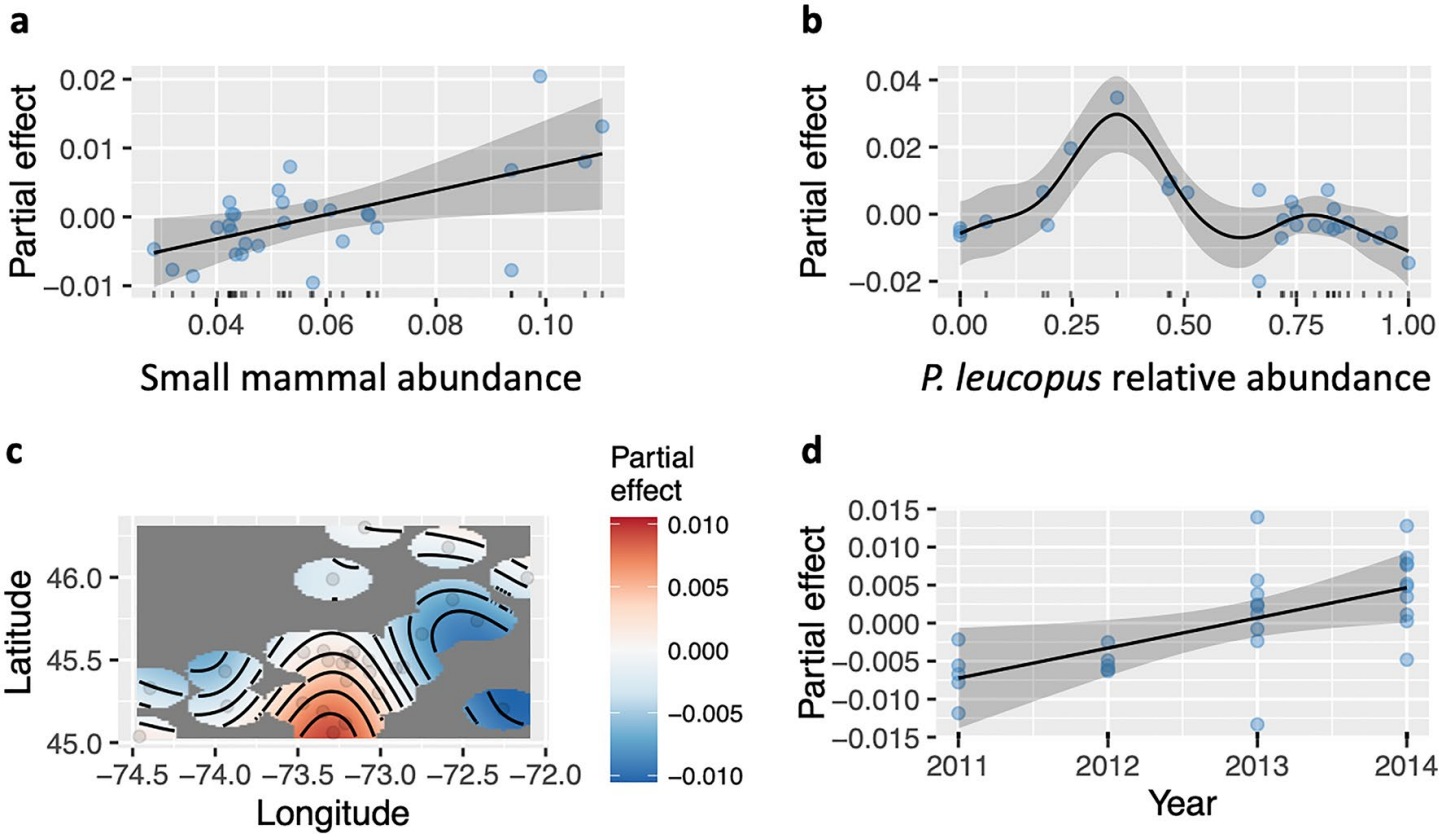
Partial effects of the generalized additive models with total infected tick abundance as a response variable, and smooth terms for the abundance of small mammals (**a**), the relative abundance of white-footed mice (**b**), the geographic location (**c**), and the year of collection (**d**). Shaded areas represent the 95% confidence intervals of the model fit and dots are the residual values. R-sq (adj.) = 0.716, Deviance explained = 83.6%.

The GAM (Generalized Additive Model) with the abundance of infected ticks (total tick abundance) as a response variable explained a substantial portion of the variance (*R-sq. (adj)* = 0.72), and the deviance explained by the model was also high (*Dev.* = 0.84, Table 1). The model revealed an increase over time in the abundance of infected ticks (Fig. 2d), and a decrease from a southern central region to more northern latitudes (Fig. 2c). We detected a significant effect of the relative abundance of white-footed mice on the abundance of infected ticks. This relation was non-linear, with a maximum predicted effect on infected tick abundance for small mammal host communities composed of 40% of white-footed mice (Fig. 2b). The abundance of infected ticks was the lowest when the white-footed mouse was absent from the host community – at the most north-eastern locations in our study area (Fig. 1), or when it made up more than 50% of the number of species we detected at a site (i.e. in species-poor communities). The abundance of infected ticks increased in a linear manner with the abundance of small mammal hosts (Fig. 2a).

**Table 1:** Generalized additive models of the effects of small mammal host abundance *s(N_mammals_index)*, the relative abundance of white-footed mouse *s(wfm.rel.abund)*, the year of collection *s(Year)*, and geographic location *s(Longitude, Latitude)* on the abundance of infected ticks in 29 forest sites in southern Quebec.

	edf	Ref.df	F	p-value
*s(N_mammals_index)*	0.9086	7	0.771	0.02195
*s(wfm.rel.abund)*	6.0281	8	4.356	0.00154
*s(Longitude,Latitude)*	3.9367	7	1.820	0.03088
*s(Year)*	0.9123	3	1.784	0.02285

R-sq.(adj) = 0.716, Deviance explained = 83.6%, n = 29.

### Small mammal hosts and prevalence of infected tick vectors

**Figure 3:**
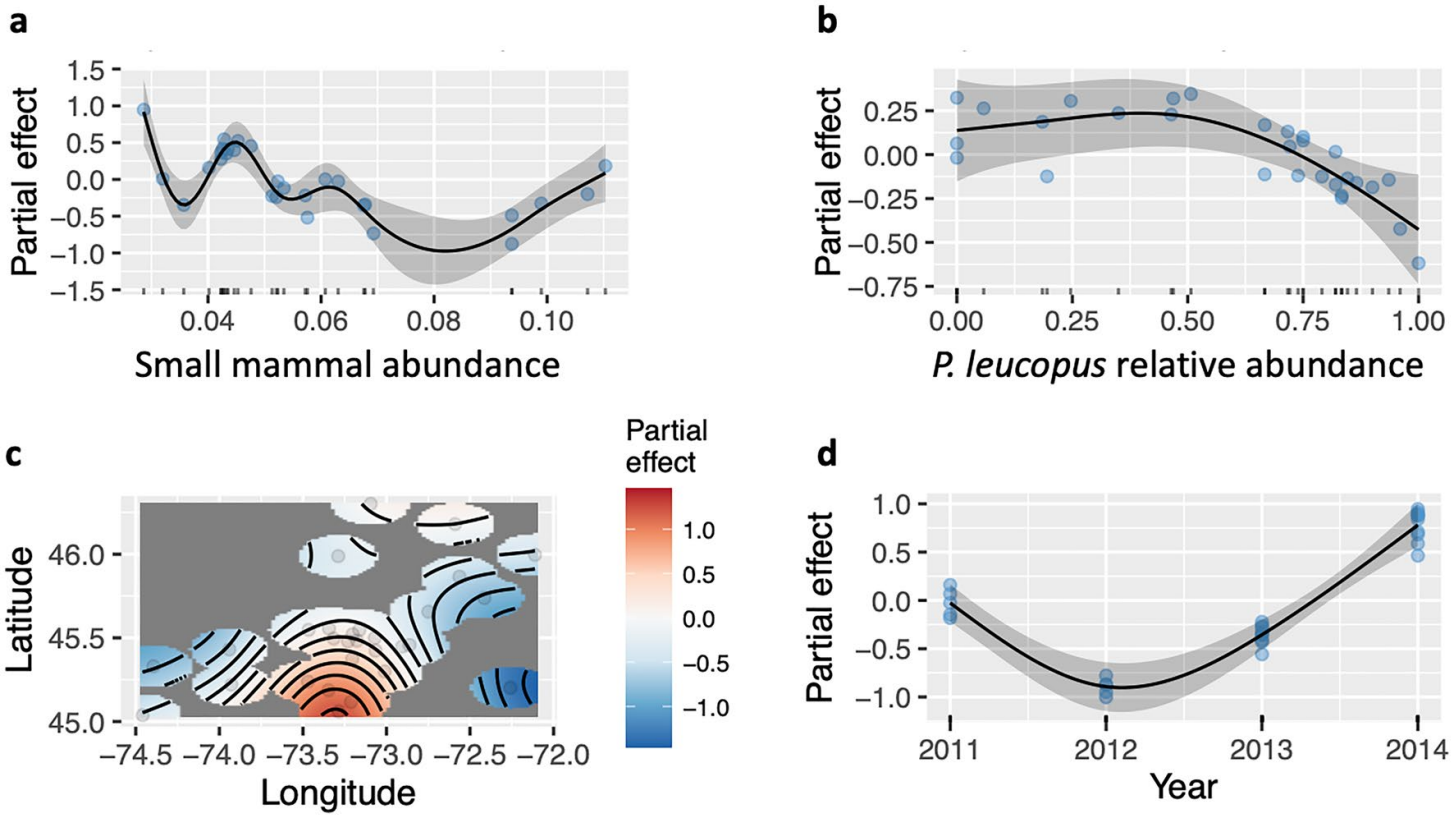
Partial effects of the generalized additive models with total infected tick prevalence as a response variable, and smooth terms for the abundance of small mammals (**a**), the relative abundance of white-footed mice (**b**), the geographic location (**c**) and the year of collection (**d**). Shaded areas represent the 95% confidence intervals of the model fit and dots are the residual values. R-sq (adj.) = 0.857, Deviance explained = 93.8%.

The GAM with the prevalence of infected ticks as a response variable explained a substantial portion of the variance (*R-sq. (adj)* = 0.86), and the deviance explained by the model was also high (*Dev.* = 0.94, Table 2). In this case again, the prevalence of infected ticks increased over time (Fig. 3d), and was larger in the most southern regions of our study area (Fig. 3c). The prevalence of infected ticks generally decreased with small mammal host abundance, up to a threshold in host abundance when infection prevalence increased (Fig. 3a). The effect of white-footed mouse abundance on infection prevalence in ticks was more consistent, with the highest infection prevalence observed in ticks when the white-footed-mouse was absent or making up a low proportion of the community (Fig. 3b).

**Table 2:** Generalized additive models of the effects of small mammal host abundance *s(N_mammals_index)*, the relative abundance of white-footed mouse *s(wfm.rel.abund)*, the year of collection *s(Year)*, and geographic location *s(Longitude, Latitude)* on the prevalence of infected ticks in 29 forest sites in southern Quebec.

	edf	Ref. df	F	p-value
*s(N_mammals_index)*	6.946	7	7.335	0.0011
*s(wfm.rel.abund)*	2.122	8	1.225	0.0229
*s(Longitude, Latitude)*	4.722	7	12.616	2.41e–05
*s(Year)*	2.000	3	17.688	3.09e–05

R-sq.(adj) = 0.857, Deviance explained = 93.8%, n = 29.

### Emergence pattern of infection prevalence with *Borrelia burgdorferi*

**Figure 4:**
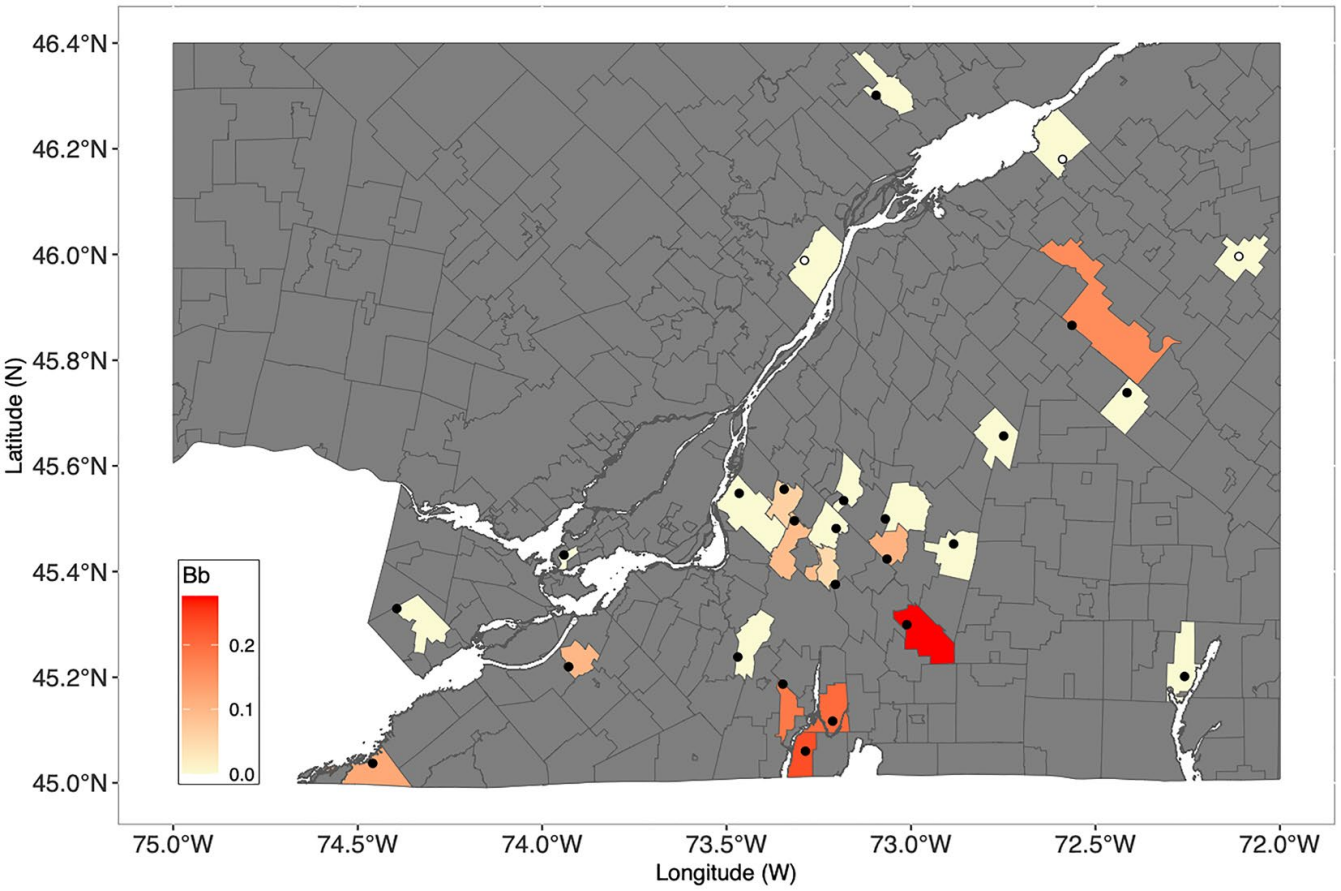
Map of the study area with the prevalence of infected ticks (Bb) displayed at the level of the sub-census division of Statistics Canada. The white-footed mouse is present at site depicted by black filled circles and was not detected at three sites represented by open circles.

The signature of the early emergence of *B. burgdorferi* s.s. was apparent both across space and over time in our data. We detected a cluster of high abundance of infected ticks and high prevalence of infection in ticks in the most southern region of our study area, below latitude 45.86 N (Fig. 4). As a corollary to this observed spatial pattern of expansion along a South-West/North-East gradient, we also confirmed a significant increase in the abundance and prevalence of *B. burgdorferi* s.s. in tick vectors from 2011 to 2014.

## Discussion

We found the signature of both amplification and dilution effects in our study area. Specifically, small mammal host communities in our study area appeared to be nested, and the white-footed mouse was the most common species across these communities, occurring even in the less species-rich communities. As expected for a dilution effect to occur, both the white-footed mouse absolute and relative abundance at a site were negatively related with the number of host species. Furthermore, *B. burgdorferi* s.s. was not detected at sites where the white-footed mouse was absent, and we detected a dilution effect whereby the prevalence of infected ticks increased with decreasing relative abundance of the white-footed mouse. However, we also detected a positive effect of small mammal host abundance on the abundance of infected ticks, supporting an amplification hypothesis^25,29,30,48–51^. Our data thus support both an amplification and a dilution effect, and we suggest that this may be a signature of an emerging disease system.

As time passes and *B. burgdorferi* s.s. prevalence continues to increase in southern Quebec, one of the effects may strengthen or some other relationship between host diversity, abundance, and disease prevalence may emerge. In fact, a high host diversity may reduce the transmission and risk of existing diseases while increasing the probability of new disease emerging^32^. Our study area in southern Quebec covers a range of risk status for Lyme disease, from high risk in the most southern part of the province, to no-risk areas in the most northern parts^52^. The white-footed mouse is currently rapidly shifting its distribution range poleward^42^. This increase in abundance and shift in distribution are expected to impact local species composition and abundance (e.g.^53^). In such a dynamic system, which varies across both space and time, it is unlikely that the host-disease relationship will remain unchanged^28^.

Our study focused on the role of *P. leucopus*, because this species is considered the most competent reservoir for *B. burgdorferi* s.s. in northeastern North America^54^. *P. leucopus* may thus play a central role in the transmission and spread of *B. burgdorferi* s.s. in southern Quebec^39^, although the spread of *B. burgdorferi* s.s. into northern latitudes may be further facilitated by the endemic deer mouse (*P. maniculatus*), which is the natural host for *Ixodes* ticks in Western North America^55^. In fact, there is evidence of hybridization occurring between the two mouse species at the northern edge of the distribution of *P. leucopus* in Quebec^44,56^. As generalist species, ticks may feed on either mouse species present at a given location and time, and the prevalence of *B. burgdorferi* s.s. at this location will thus depend on the competence and relative abundance of the two hosts.

Detecting a relationship between host diversity or abundance and pathogen prevalence is not enough to identify the mechanisms driving this relationship. Few empirical studies have tested for a relationship between biodiversity and *B. burgdorferi* s.s. prevalence in Lyme disease vectors and hosts^24,32,34^, and no study has subsequently directly investigated the mechanisms driving the observed relationship in the Lyme disease system in North America. Numerous mechanisms have been proposed which could lead to an amplification effect of biodiversity on *B. burgdorferi* s.s.^26^, and many have suggested that vector amplification could play an important role^26,28,29^, especially for emerging pathogens^30^. The positive relationship between host abundance and *B. burgdorferi* s.s. prevalence we observed does not preclude the possibility that some mechanisms consistent with dilution are taking place along with amplifying mechanisms. Here, we observed a dilution effect, but only up to a maximum relative abundance of *P. leucopus* within the host community. Beyond this threshold, the net effect of hosts on disease prevalence is an amplification effect. Similarly, using a multi-species model, Mihaljevic et al.^50^ predicted that the diversity-disease relationship is neither linear, nor monotonic. They concluded that an amplification may occur below a threshold of species richness, above which a dilution effect takes place, a theoretical prediction supported by our empirical data.

Our observation of an increased prevalence of infected ticks from 2011 to 2014 is consistent with early-stage emergence of Lyme disease in southern Quebec (see also^38,39,57^. Much of the previous work on the diversity-*B. burgdorferi* s.s. relationship has taken place in the northeast United States, and findings and theory based on these studies may not be applicable in other regions. For instance, in areas where Lyme disease is emerging, the dispersal of tick-carrying migratory birds into a given habitat, and the stage of establishment of a new tick population, may play a larger role than host community composition in determining the prevalence of *B. burgdorferi* s.s. at said site^38,58–60^. As Lyme disease establishes in southern Quebec, the relationships between small mammal communities, ticks and *B. burgdorferi* s.s. communities may begin to resemble those hypothesized by Ostfeld and Keesing^30^ for areas where the disease is endemic. Our study is the first to provide empirical data supporting the hypothesis that an amplification effect may be more prevalent in emerging disease systems, compared to areas of endemism where a dilution effect is modulating infection prevalence. We predict that the host diversity-disease relationship will strengthen over time, as *B. burgdorferi* s.s. continues to emerge in southern Quebec^61^.

*B. burgdorferi* s.s. was absent at the three sites where white-footed mice were absent. This suggests that the presence of this host species may be essential for efficient transmission of *B. burgdorferi* s.s. in the region. Locations where *B. burgdorferi* s.s. is efficiently transmitted are thus expected to expand northward in this region^39^, as the range of white-footed mice moves northward under climate change^41,43^.

We found that the prevalence of infected ticks is greatest in the southern-most part of our study area, close to the US border. The plausible mechanisms for this are twofold and interrelated. First, the range of tick vectors and *B. burgdorferi* s.s. is moving northwards due to climate change^23,38,39,46^. We would thus expect that the abundance of ticks and *B. burgdorferi* s.s. are greater at more southern latitudes, as well as the degree of establishment of tick populations^59^. Second, at low latitudes, warmer temperatures may generate habitats with conditions more amenable to white-footed mice^41^, other small mammals and ticks^42,46^. Our results support a role of climate change in Lyme disease emergence in southern Quebec, as other authors have concluded^38,39,42^, and climate warming is expected to accelerate the increase in incidence of Lyme disease in Quebec, elsewhere in Canada^23,62^, and globally^63^.

The monitoring of range expansion of tick vectors and small mammal hosts under climate change in Canada, will continue to play an important role in understanding and predicting the emerging pattern of ecological disease risk. Of further concern is the increasing number of human disease pathogens borne by *I. scapularis* described over the past decade^64^, and the common occurrence of co-infection among these pathogens^65^. In a context of rapid global change, with shifts in species distribution, and turnover in local community composition, there is an urgent need for a better understanding of the local dynamics of host/vectors/pathogens interactions to forecast future disease outbreaks.

Our work emphasizes the importance of conducting studies of northern range shifts for understanding and predicting Lyme disease risk. We found evidence that the composition of the small mammal communities is an important modulator of ecological Lyme disease risk locally. We observed both an amplification effect of small mammal species abundance in communities with the highest abundance of mammal hosts, as well as a dilution effect of the relative abundance of white-footed mouse. We also uncovered the important role of regional processes (i.e. distribution shifts of host and vector species) in modulating the rate and pattern of emergence of Lyme disease locally. Therefore, in invasion/emergence events for wildlife diseases, whether a dilution or amplification effect occurs is time/invasion stage-dependent, not just dependent on geographic scale, as has been suggested^18^.

One limitation of our work is the relatively limited time covered by our sampling period (2011–2014), which only captures the early stages of emergence of Lyme disease in our study area. Our study was also focused on Southern Quebec, a region of Southern Canada bounded to the West by the Great Lakes and crossed in a near west–east direction by the St-Lawrence, a major water barrier to animal dispersal. Further work with larger temporal and geographical scopes will allow to test the generality of our findings, but more importantly, to increase our ability to assess the timing of the switch of this disease system from early emergence to establishment. Based on our results, we argue that the nature of the relationship between biodiversity and disease risk is strongly time dependent during the phase of emergence and spread. Therefore, to be reliable, disease risk predictions based on future host distribution patterns under climate warming should first identify the degree of establishment, or time since first occurrence, of a pathogen.

## Materials and methods

### Field methods

We sampled twenty-nine sites inside patches of deciduous or mixed deciduous-coniferous forest in southern Quebec from 2011 to 2014. The study sites were located in a region around the city of Montreal measuring approximately 184 by 140 km, or 25,868 km^2^, characterized by agriculture, urban development, and forests (Fig. 1)^66^.

In each forest patch, grids of Sherman traps were set for two to three consecutive nights. Depending on trapping success, sampling effort varied across sites, and the number of trap-nights (number of traps x number of trapping nights) ranged from 112 to 1120 (Table S1). The traps were freshly baited each night and checked the following morning. Trapped mammals were euthanized in situ with an overdose of isoflurane inhalation performed in a closed chamber followed by cervical dislocation, in compliance with the guidelines for rodent euthanasia of the Canadian Council on Animal Care (https://ccac.ca/).

Small mammals individuals were examined under a dissecting microscope and ticks were removed and preserved in 95% ethanol. Nymphs and adults were kept in individual sample tubes, while all larvae collected from a single host were pooled into one sample tube. Individuals of *Peromyscus leucopus* and *P. maniculatus,* were identified to the species level using species-specific primers of DNA extracted from liver tissue, as described in^67^. In addition to collecting ticks from the trapped mammals, we sampled ticks by dragging 1 m^2^ flannel sheets along the forest floor. The dragging transects followed the length of the trapping grids and the dragging distance per site ranged from 360 to 1440 m. The sheets were examined for ticks every 10 m of dragging along each transect. The abundance of ticks at each study site was estimated as the sum of the ticks collected by dragging and from trapped mammals. All ticks collected were preserved in 95% ethanol. Nymphs and adults were kept in distinct individual sample tubes, while all larvae collected from the drag between each stop along each transect were pooled into one tube.

All mammals, nymph and adult ticks collected from dragging as well as all ticks from trapped mammals were screened for the presence of *B. burgdorferi* s.s. at the National Microbiology Laboratory of the Public Health Agency of Canada. DNA was extracted from ticks and mammal tissues (heart) and screened for *B. burgdorferi* s.s. using real-time polymerase chain reactions targeting the genus-specific 23S ribosomal RNA and species-specific *ospA* genes as described in^68^.

### Statistical analyses

All analyses were performed in R version 4.2.2^69^. Because a nested pattern in host community composition is a necessary condition for a dilution effect to occur^28^, we first tested for patterns in host species occurrence across our sites using the function *nestedtemp* in the *vegan* package^70^. We evaluated if small mammal communities were nested across our study sites, and if so, whether the most common small mammal species (i.e. the species present at all sites, including in those with the lowest number of coexisting host species) was also the most efficient reservoir host (i.e. the white-footed mouse in our system). To evaluate whether the most common host species was also the most prevalent across our sites, we calculated the community composition (sites x species) “temperature” of our host community data, with matrix temperature increasing with community nestedness, and tested its significance against a set of 999 random communities sampled from our original community matrix^71^.

Sampling effort was measured by the number of trap nights (for small mammals), number of small mammals captured (for feeding ticks), or dragging distance (for questing ticks). Because the sampling effort varied across our study sites, we estimated the abundance of small mammals and ticks with an index of abundance as the total count over sampling effort (trap-nights for small mammals and dragging distance for ticks). The prevalence of infected ticks was estimated by the number of infected ticks over the total number of ticks sampled at a site.

We ran two generalized additive models using the *mgcv* package in R^72^. We first aggregated the data at the site level, by calculating either the sum (measures of abundance) or the maximum (number of host species, year) of each variable. Small mammals could be acting as more effective “collectors” of ticks and feeding tick larvae collected from mammals could have acquired infection while feeding, while questing larvae are universally uninfected^73,74^. We thus calculated the correlation between feeding and questing larvae abundance and infection prevalence to check for the absence of an effect of sampling method in our tick data. We then explored the relationships between host mammal species richness and their abundance, and the absolute and relative abundance of white-footed mice at a site, and selected only the variables that were not significantly correlated with the other variables to include in the GAMs. We tested for spatial autocorrelation in our data using the Moran I statistics calculated in the *ape* package^75^, and an interaction smooth term *s*(Longitude, Latitude) was included in all models. Other variables included in each model were the abundance of small mammal hosts (the number of trapped small mammals corrected for sampling effort), the relative abundance of *P. leucopus* (the proportion of trapped *P. leucopus*) and the collection year (the most recent year, when a site was sampled several years).

### Ethics declaration

All procedures performed were approved by the McGill Center for Animal Research Ethics (McGill AUP#5420) and the government of Quebec (SEG permits 2011-05-15-014-00-S-F, 2012-07-16-1417-16-17-SF, 2013-07-04-1530-04-14-16-17-SF, and 2014-05-02–1638-05-16-SF), and data reported in this work follow the ARRIVE guidelines 2.0 (https://arriveguidelines.org/arrive-guidelines).

### Supplementary Information

Supplementary Information.

## Data Availability

All data used for this study are included in this article as Supplementary Information files.
