# Supplementary material for: It’s about time: small mammal communities and Lyme disease emergence
Source: Sci Rep. 2023 Sep 4;13:14513. doi: 10.1038/s41598-023-41901-z (PMC10477272; doi:10.1038/s41598-023-41901-z)

Supplementary Information

It's about time: Small mammal communities and Lyme disease emergence

V. Millien[1, 2], S. S. T. Leo[1, 2], S. Turney[1, 2], A. Gonzalez[2]

[1] Redpath Museum, McGill University, Montréal, QC, H3A 0C4, Canada

[2] Department of Biology, McGill University, Montréal, QC, H3A 1B1, Canada

Corresponding author: Virginie Millien, Redpath Museum, McGill University, Montréal, QC, H3A 0C4, Canada, Tel: 1 (514) 398-4849, Fax: 1 (514) 398-4065, E-mail: virginie.millien@mcgill.ca

Table S1. Latitude and longitude of all study sites, the number of trap nights and total dragging distance in meters, the total number of trapped small mammal hosts and *Peromyscus leucopus*, the total number of ticks and infected ticks (collected by dragging or removed from trapped hosts). 1: Austin; 2: Base Militaire Farnham; 3: Boat_Burmese; 4: Drumondville; 5: Grand Bois; 6: Henryville; 7: Horse Ranch; 8: Jurassic Park; 9: Killer Dog; 10: Lefbvre; 11: Logging Site; 12: Longueuil Parc; 13: Mont Rougemont; 14: Mont St. Bruno; 15: Mont St. Hilaire; 16: Mont Yamaska; 17: Morgan Arboretum; 18: Nicolet; 19: Noyan; 20: Orchad; 21: Parc des Chutes Ste Ursule; 22: Reserve St Francois-du-Lac; 23: Reserve Tourbieres de Lanoraie; 24: St-Albert; 25: St Etienne de Beauharnois; 26: Saint-Jacques le Mineur; 27: Saint Liboire; 28: Saint Polycarpe; 29: Saint Valentin.

					small mammals		feeding ticks		questing ticks	
**Site**	**Latitude (N)**	**Longitude (W)**	**trap nights**	**dragging distance (m)**	**total**	**white-footed mice**	**total**	**infected**	**total**	**infected**
1	45.20	-72.26	1136	3720	69	4	1	0	3	0
2	45.30	-73.01	472	2100	20	7	29	7	91	26
3	45.48	-73.23	672	1440	30	25	3	0	0	0
4	45.87	-72.56	1360	4380	150	37	71	13	36	4
5	45.38	-73.21	896	960	36	27	21	0	19	2
6	45.12	-73.21	472	2100	32	15	21	4	53	11
7	45.50	-73.32	1704	2580	73	37	30	5	36	1
8	45.52	-73.20	1148	1320	52	44	4	0	0	0
9	45.48	-73.18	896	1440	39	32	5	0	2	0
10	45.74	-72.41	712	2040	41	8	9	0	0	0
11	45.45	-72.91	896	960	46	34	15	0	17	0
12	45.55	-73.47	592	2400	41	41	11	0	1	0
13	45.50	-73.07	1008	1440	43	31	4	0	2	0
14	45.56	-73.34	936	2700	50	41	50	5	63	2
15	45.55	-73.16	252	360	12	9	0	0	0	0
16	45.46	-72.86	784	960	28	13	2	0	8	0
17	45.43	-73.94	480	1440	25	24	1	0	0	0
18	46.18	-72.59	224	960	21	0	1	0	0	0
19	45.06	-73.29	576	3060	57	38	28	6	28	7
20	45.42	-73.07	1816	2580	52	45	8	0	11	2
21	46.30	-73.09	1344	2880	43	34	0	0	0	0
22	45.04	-74.46	832	3540	36	30	22	3	19	2
23	45.99	-73.29	112	960	12	0	0	0	0	0
24	46.00	-72.11	544	2280	23	0	1	0	0	0
25	45.22	-73.93	952	3540	60	43	10	1	10	1
26	45.24	-73.47	352	2100	33	22	6	0	5	0
27	45.66	-72.75	592	2400	40	36	0	0	1	0
28	45.33	-74.39	592	2400	31	29	0	0	0	0
29	45.19	-73.35	472	2100	27	5	13	5	63	9

Table S2. Number of small mammals captured at all study sites. 1: Austin; 2: Base Militaire Farnham; 3: Boat_Burmese; 4: Drumondville; 5: Grand Bois; 6: Henryville; 7: Horse Ranch; 8: Jurassic Park; 9: Killer Dog; 10: Lefbvre; 11: Logging Site; 12: Longueuil Parc; 13: Mont Rougemont; 14: Mont St. Bruno; 15: Mont St. Hilaire; 16: Mont Yamaska; 17: Morgan Arboretum; 18: Nicolet; 19: Noyan; 20: Orchad; 21: Parc des Chutes Ste Ursule; 22: Reserve St Francois-du-Lac; 23: Reserve Tourbieres de Lanoraie; 24: St-Albert; 25: St Etienne de Beauharnois; 26: Saint-Jacques le Mineur; 27: Saint Liboire; 28: Saint Polycarpe; 29: Saint Valentin.

Site	Blarina brevicauda	Sorex cinereus	Sorex fumeus	Myodes gapperi	Zapus hudsonius	Napaeozapus insignis	Peromyscus leucopus	Peromyscus maniculatus	Tamias striatus
1	11	1	1	7	0	13	4	30	2
2	0	0	0	3	0	3	7	6	1
3	3	1	0	0	0	0	25	1	0
4	3	1	0	104	4	0	37	1	0
5	7	0	0	0	0	2	27	0	0
6	3	1	0	3	0	0	15	10	0
7	5	27	0	0	0	1	37	0	3
8	4	0	0	2	0	1	44	0	0
9	2	5	0	0	0	0	32	0	0
10	6	0	0	5	0	1	8	21	0
11	6	0	0	5	0	0	34	1	0
12	0	0	0	0	0	0	41	0	0
13	7	0	1	2	0	0	31	1	1
14	2	3	0	1	0	0	41	0	2
15	3	0	0	0	0	0	9	0	0
16	2	0	0	0	0	0	13	13	0
17	1	0	0	0	0	0	24	0	0
18	1	0	0	16	0	0	0	4	0
19	2	2	0	9	0	0	38	6	0
20	6	1	0	0	0	0	45	0	0
21	3	1	0	0	0	0	34	1	0
22	3	1	0	0	1	0	30	0	1
23	1	0	0	2	0	0	0	8	0
24	1	0	1	11	0	4	0	4	1
25	10	1	0	0	0	0	43	4	2
26	3	3	0	1	0	0	22	4	0
27	3	0	0	0	0	0	36	0	1
28	2	0	0	0	0	0	29	0	0
29	3	3	0	15	0	0	5	1	0

Figure S1. Small mammal community nestedness analysis. Left: Site x species incidence diagram; matrix fill is 45%, and site fill ranges from 0 to 96%. Right: Temperature for 999 simulated site x species community matrices and observed temperature (red line). The observed temperature is 18.30, and only 30% of the simulated community are more nested (colder).

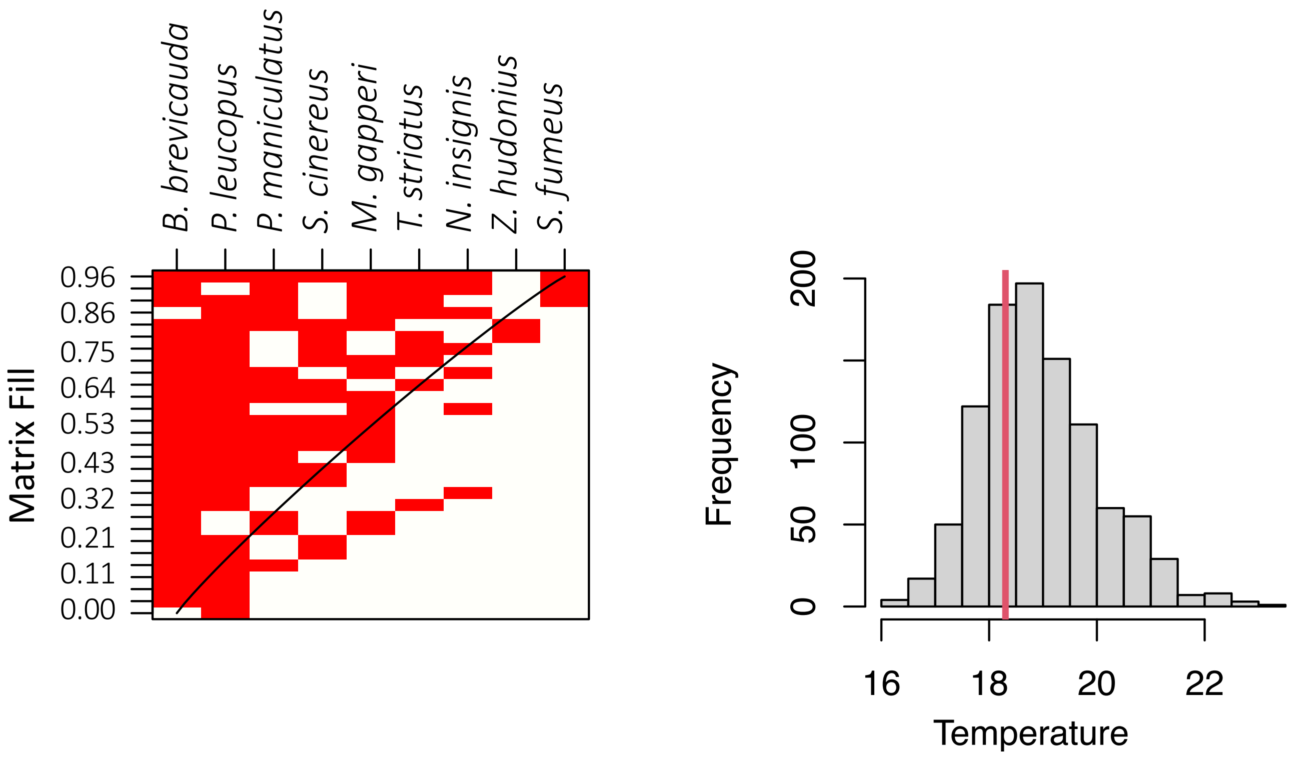

Figure S2. Spatial variation in the relative abundance of white-footed mice (*Moran I* = 0.04, $p < 0.03$), the abundance of small mammals corrected by sampling effort (*Moran I* = 0.05, $p < 0.02$), the prevalence of infected feeding ticks (*Moran I* = 0.09, $p < 0.04$) and the prevalence of infected questing ticks (*Moran I* = 0.05, $p = 0.12$) in Southern Quebec. Values are those observed at the site level and displayed at the level of the sub-census division of Statistics Canada.

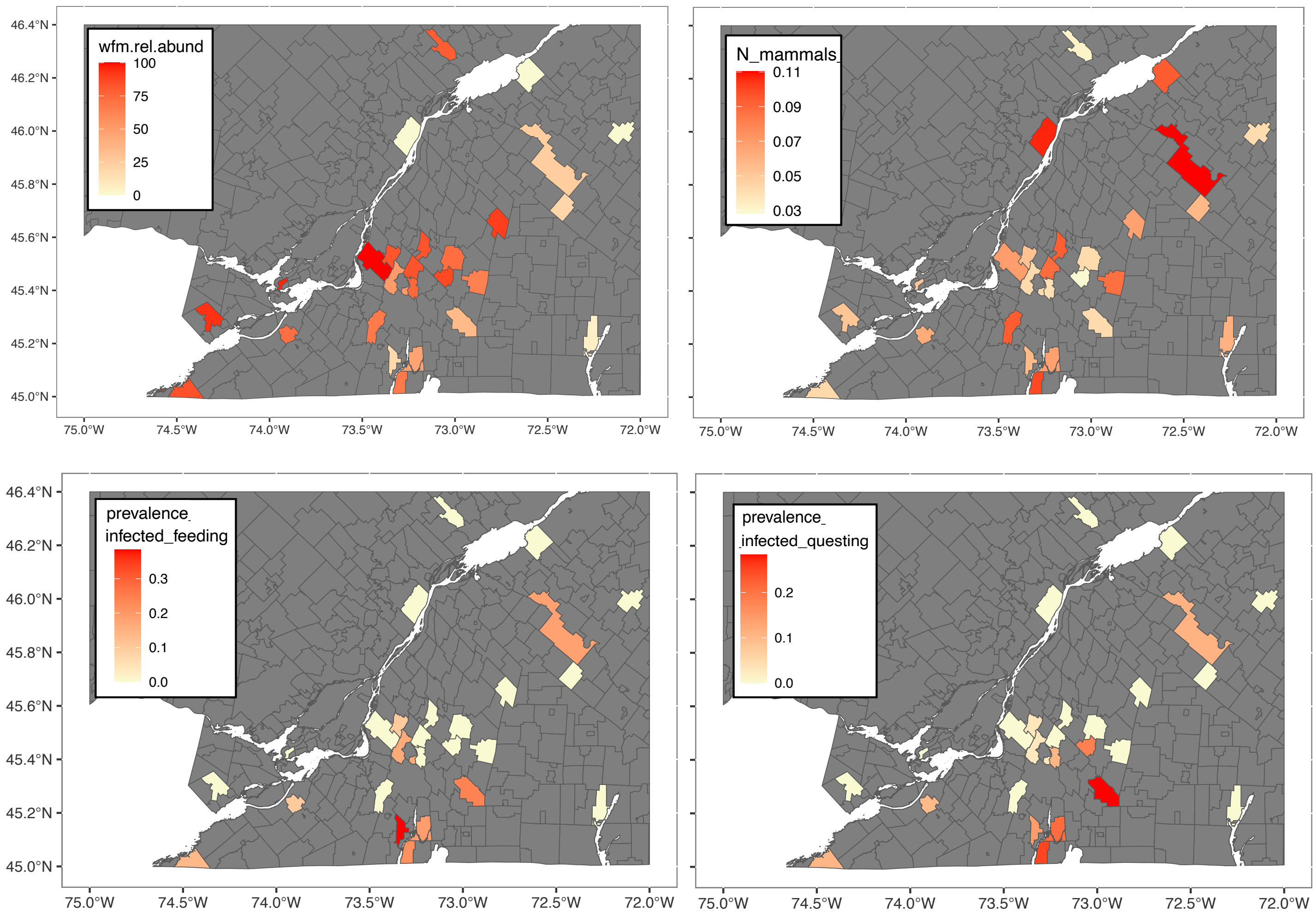

Supplement: Supplementary file 1 — Supplementary Information. [file 41598_2023_41901_MOESM1_ESM.pdf]
